# Retraction: Tocilizumab inhibits neuronal cell apoptosis and activates STAT3 in cerebral infarction rat model

**DOI:** 10.17305/bb.2025.12568

**Published:** 2025-05-07



Shaojun Wang, Jun Zhou, Weijie Kang, Zhaoni Dong, Hezuo Wang

Vol. 16 No. 2 (2016) | http://dx.doi.org/10.17305/bjbms.2016.853 | January 15, 2016

Following publication, concerns were raised by readers and the public regarding the integrity of the data presented in two figures of this article:

**Figure 2B:** An unexpected overlap was found between an image presented in this study and figures published in other articles [1, 2, 3, 4, 5, 6, 7, 8].

**Figure 4:** An unexpected overlap was detected within the Western blot panels, suggesting potential duplication or manipulation.

The editorial office contacted all authors on 13 January 2025 and 30 January 2025 to clarify these concerns. On 4 March 2025, the corresponding author was specifically approached through both the e-mail address provided upon article submission and an institutional e-mail located online. However, no response or explanation was ever received.

Following our journalʼs policies and guidelines from the Committee on Publication Ethics (COPE), the editors have decided to retract the article from publication due to serious concerns about the reliability and originality of the data. This retraction is intended to maintain the integrity of the scientific record.

We apologize to the readership for any inconvenience caused. We thank the individuals who brought these concerns to our attention.

The Editor-in-Chief has approved this retraction note.

## References

[1] Wang S, Zhou J, Kang W, Dong Z, Wang H (2016). Tocilizumab inhibits neuronal cell apoptosis and activates STAT3 in cerebral infarction rat model. Biomol Biomed.

[2] Zhu K, He Y, Xia C, Yan W, Song T, Zhang B (2016). MicroRNA-15a inhibits proliferation and induces apoptosis in CNE1 nasopharyngeal carcinoma cells [retracted in: Oncol Res. 2024;32(10):1683-1684]. Oncol Res..

[3] Yan D, Cai X, Feng Y (2016). miR-183 modulates cell apoptosis and proliferation in tongue squamous cell carcinoma SCC25 cell line [retracted in: Oncol Res. 2024;32(10):1691-1692]. Oncol Res..

[4] Pan C, Wang D, Zhang Y, Yu W (2016). MicroRNA-1284 inhibits cell viability and induces apoptosis of ovarian cancer cell line OVCAR3. Oncol Res..

[5] Huang W, Lan X, Li X, Wang D, Sun Y, Wang Q (2017). Long non-coding RNA PVT1 promotes LPS-induced septic acute kidney injury by regulating TNFα and JNK/NF-κB pathways in HK-2 cells. Int Immunopharmacol..

[6] Cao B, Liu C, Yang G (2018). RETRACTED: Down-regulation of lncRNA ADAMTS9-AS2 contributes to gastric cancer development via activation of PI3K/Akt pathway. Biomed Pharmacother..

[7] Liu Y, Song Y, Zhu X (2017). MicroRNA-181a regulates apoptosis and autophagy process in Parkinson's disease by inhibiting p38 mitogen-activated protein kinase (MAPK)/c-Jun N-terminal kinases (JNK) signaling pathways. Med Sci Monit..

[8] Zheng J, Lin Z, Zhang L, Chen H (2016). MicroRNA-455-3p inhibits tumor cell proliferation and induces apoptosis in HCT116 human colon cancer cells. Med Sci Monit..

